# Drug-Repurposing Screening Identifies a Gallic Acid Binding Site on SARS-CoV-2 Non-structural Protein 7

**DOI:** 10.1021/acsptsci.2c00225

**Published:** 2023-03-07

**Authors:** Yushu Gu, Miaomiao Liu, Bart L. Staker, Garry W. Buchko, Ronald J. Quinn

**Affiliations:** † Griffith Institute for Drug Discovery, Griffith University, Brisbane 4111, Australia; $ Seattle Children’s Research Institute, Seattle, Washington 98101, United States; ‡ Earth and Biological Sciences Directorate, Pacific Northwest National Laboratory, Richland, Washington 99354, United States; + School of Molecular Biosciences, Washington State University, Pullman, Washington 99164, United States

**Keywords:** COVID-19, SARS-CoV-2, native mass spectrometry, drug repurposing, antiviral

## Abstract

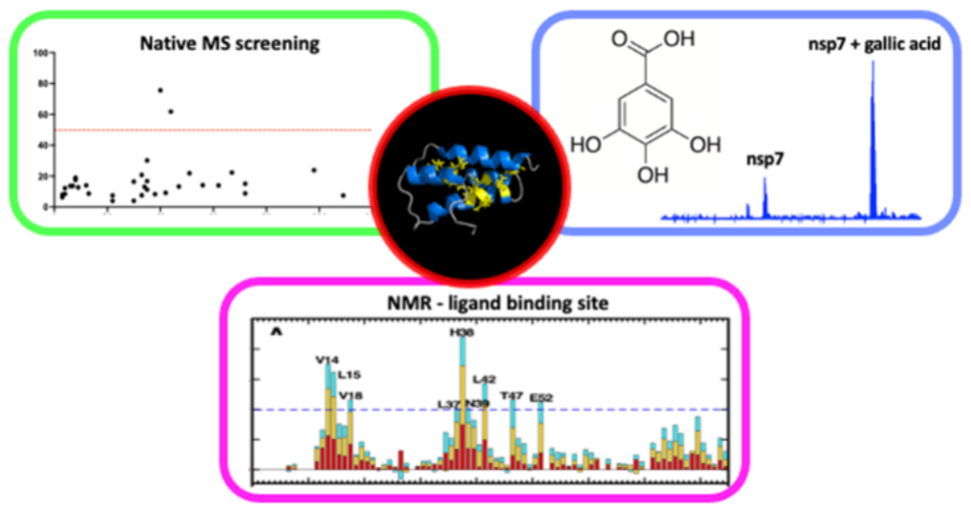

SARS-CoV-2 is the agent responsible for acute respiratory disease COVID-19 and the global pandemic initiated in early 2020. While the record-breaking development of vaccines has assisted the control of COVID-19, there is still a pressing global demand for antiviral drugs to halt the destructive impact of this disease. Repurposing clinically approved drugs provides an opportunity to expediate SARS-CoV-2 treatments into the clinic. In an effort to facilitate drug repurposing, an FDA-approved drug library containing 2400 compounds was screened against the SARS-CoV-2 non-structural protein 7 (nsp7) using a native mass spectrometry-based assay. Nsp7 is one of the components of the SARS-CoV-2 replication/transcription complex essential for optimal viral replication, perhaps serving to off-load RNA from nsp8. From this library, gallic acid was identified as a compound that bound tightly to nsp7, with an estimated *K*_d_ of 15 μM. NMR chemical shift perturbation experiments were used to map the ligand-binding surface of gallic acid on nsp7, indicating that the compound bound to a surface pocket centered on one of the protein’s four α-helices (α2). The identification of the gallic acid-binding site on nsp7 may allow development of a SARS-CoV-2 therapeutic via artificial-intelligence-based virtual docking and other strategies.

The global COVID-19 pandemic has taken over 6 million lives worldwide as of August 2, 2022.^1^ Due to the emerging variants of SARS-CoV-2, the agent responsible for this acute respiratory disease, modified vaccines are required to keep pace with the virus’s evolving gene products. Currently, remdesivir (aka Veklury) and molnupiravir, RNA-dependent RNA polymerase inhibitors; baricitinib, a Janus kinase inhibitor; and nirmatrelvir/ritonavir, nsp5 proteases inhibitors, are the only small-molecule drugs approved by the U.S. Food and Drug Administration (FDA) for the treatment of COVID-19.^2−7^ There have been mixed reports of the efficacy of remdesivir on treating COVID-19, with the WHO Solidarity trial that included remdesivir showing it had no significant effect on reducing mortality, though the outcomes of other trials are yet to be published.^8,9^ A combination treatment of baricitinib and remdesivir showed higher efficacy than remdesivir alone in reducing recovery time for patients with SARS-CoV-2.^10^ Molnupiravir should not be used by children or pregnant persons, and the nirmatrelvir/ritonavir combination may cause significant interactions for patients taking other drugs.^11^ Because clinical deployment of new SARS-CoV-2 therapeutics is still needed, repurposing drugs already approved for different purposes avoids the time-comsuming process of developing and approving new antiviral therapeutics. For example, sildenafil, a cyclic guanosine monophosphate (cGMP)-specific phosphodiesterase type-5 inhibitor, was initially introduced as an antianginal drug and eventually repurposed into a drug for treating erectile dysfunction.^12,13^ Other successful drug repurposing examples include itraconazole, nelfinavir, and thalidomide.^14−17^

SARS-CoV-2, a member of the genus *Betacoronavirus*, is a single-stranded positive sense RNA virus with a large genome (∼30 000 bp) in comparison to most other RNA viruses.^18,19^ Its genome contains two large opening reading frames (ORFs), ORF1a and ORF1b, which encode 16 non-structural proteins (nsp’s).^20^ The remaining ORFs at the 3′ end of the genome contain four structural proteins—spike (S), envelope (E), membrane (M), and nucleocapsid (N)—as well as at least nine putative accessory factors (ORFs).^21−23^ The nsp’s assemble into a multisubunit complex which mediates SARS-CoV-2 viral replication and transcription.^24^ The catalytic core of this complex is the RNA-dependent RNA polymerase (RdRp) domain, present in nsp12, that is essential for viral RNA synthesis.^25,26^ Nsp12 is the target of the prodrug remdesivir, which is transformed into its active form, remdesivir triphosphate, after it enters cells, where it is covalently incorporated into the primer strand of replicating viral RNA, terminating chain elongation.^27^ Alone, nsp12 is capable of RNA replication; however, it displays minimal polymerase activity and requires association with nsp7 and nsp8 for maximal RNA synthesis efficiency.^28^ Nsp7 is an ∼9 kDa protein that, alone, self-assembles into multimers in solution and does not interact with RNA.^24^ In the presence of nsp8, nsp7 will form transient complexes with nsp8 and together with nsp12, nsp13, and nsp9, establishing the viral replication and translation complex (RTC) that associates with RNA.^29−35^ While the RTC is dynamic, cryo-EM structures have been solved for stable 1:2:1:1 nsp7:nsp8:nsp12:RNA(1× or 2×) complexes.^35^ The precise role of nsp7 in the RTC is still unclear, but, because of its affinity for nsp8 and lack of affinity for RNA, it has been suggested that its role may be to off-load RNA from nsp8.^34^ Regardless of the mechanism, nsp7 is vital for optimal RNA synthesis by the viral RTC, and any ligand that binds to nsp7 and disrupts the RTC may have therapeutic potential.

This study focuses on screening an FDA-approved library containing 2400 compounds against nsp7 using a native mass spectrometry (native MS)-based assay. Native MS is a label-free, robust method with the advantage of direct observation of protein–ligand binding without disrupting non-covalent interactions.^36−39^ Consequently, the biological functionality of the analyte molecules can be well reflected. In native MS, the molecular weight (MW) of the bound ligand can be determined directly by using the difference between the mass-to-charge ratios (*m*/*z*) of the unbound protein ions and the protein–ligand complex ions multiplied by its charge state.^37^ The high sensitivity of this technique allows the detection of fragment hits with low affinity, up to 1 mM.^38,40^ Our group has successfully used native MS to identify ligands from natural product or compound libraries that bind to specific target proteins.^40−44^ This success includes the identification of oridonin from an in-house natural product library that selectively bound to the SARS-CoV-2 protein nsp9.^44^

Here we report the use of native MS to identify gallic acid, out of an FDA-approved library of 2400 compounds, that bound to the SARS-CoV-2 protein nsp7. The dissociation constant (*K*_d_) of gallic acid binding to nsp7 was determined. An NMR chemical shift perturbation study on nsp7 with gallic acid allowed us to map the ligand-binding surface of gallic acid on the structure of nsp7.

## Results and Discussion

### Native Mass Spectrometry Screen of an FDA-Approved Compound Library against SARS-CoV-2 nsp7

**Figure 1:**
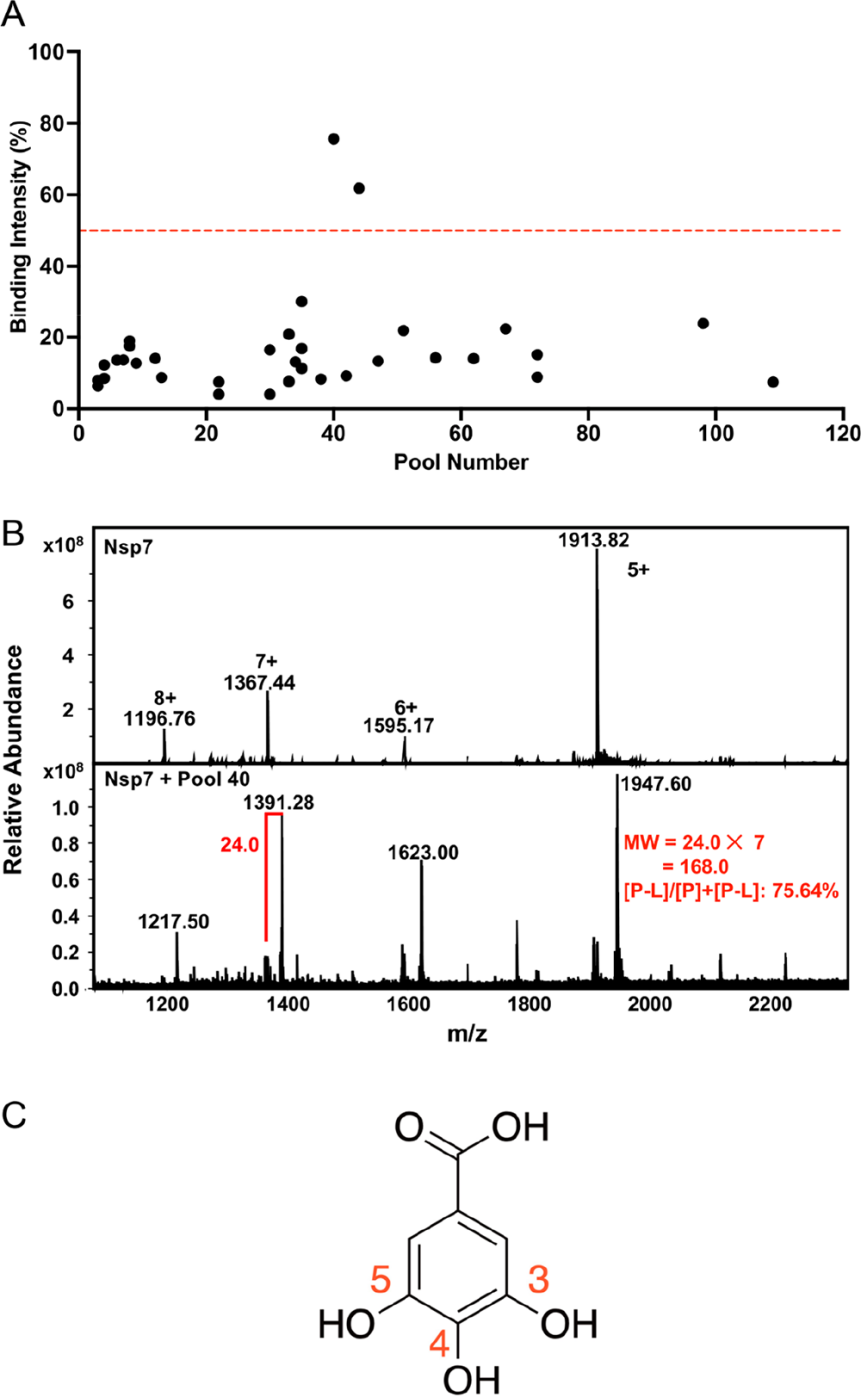
**Native MS screening of an FDA-approved drug library against SARS-CoV-2 nsp7.** (**A**) Cumulative results of initial library screening of the 120 pools of 20 FDA-approved drugs against nsp7. Red line indicates the cutoff binding threshold, an *m*/*z* intensity >50%. (**B**) Overlay of the native mass spectra of free nsp7 (top) and nsp7 incubated with FDA Pool 40. (**C**) Chemical structure of gallic acid, the compound identified as the ligand bound to nsp7 from Pool 40.

SARS-CoV-2 nsp7 was screened against a compound library of 2400 FDA-approved drugs to identify small-molecule binders. Each pool in the library contained 20 small molecules with a range of molecular weights for unambiguous identification. As shown in Figure 1A, 34 out of 120 pools showed detectable ligand binding, with two of the pools having binding activity with intensity greater than 50%, giving a hit rate of ∼0.1% overall. A high-resolution electrospray ionization Fourier transform ion cyclotron resonance mass spectrometer (ESI-FT-ICR-MS) was used for the native MS experiments. Nsp7 appeared as charge states +5 to +8 (Figure 1B, top spectrum). The carbon isotope patterns (SI Figure S1) confirmed that all peaks were due to a monomer. Previous NMR conformational studies showed two major forms of nsp7, with one form containing a rotation of helix α4 away from the α2/α3 core and helical folding of the polypeptide segment of residues 3–12, and the other showing tight binding of α2/α3/α4.^45^ Based on this information, it is likely that the native MS shows major species at +7 (+6 to +8 triad) and +5. The bottom spectrum in Figure 1B shows nsp7 and Pool 40. It demonstrated strong binding to all charge state species. The most strongly bound ligand was from Pool 40, with MW = 168.0 Da (Figure 1B). This molecular weight closely matched to gallic acid (MW = 170.1 Da), one of the 20 compounds in this pool. Gallic acid is a trihydroxybenzoic acid with three hydroxy groups at aromatic ring positions 3, 4, and 5 (Figure 1C).^46^ It is a phytochemical known for its antioxidant, antibacterial, and anti-inflammatory activities.^47−49^ Because of its antioxidant and free-radical-scavenging abilities, gallic acid may play a preventive role against oxidative-stress-related diseases such as cancer, degenerative diseases, and metabolic diseases.^50,51^ Gallic acid has also been shown to exhibit antiviral properties against a number of viruses, such as herpes simplex virus type 1 and human immunodeficiency virus.^52,53^ A molecular docking study with gallic acid and its derivatives against SARS-CoV-2 nsp5 protease suggested that these compounds warranted more attention for drug development.^54^ Gallic acid and its derivatives were previously examined in a molecular docking study for their potential to bind to SARS-CoV-2 nsp3, nsp5, nsp12, nsp13, and nsp15, but no *in silico* or *in vitro* study has been performed to date on the ability of gallic acid to bind to SARS-CoV-2 nsp7.^55^

### Dissociation Constant (*K*_d_) Determination of Gallic Acid with SARS-CoV-2 Nsp7

**Figure 2:**
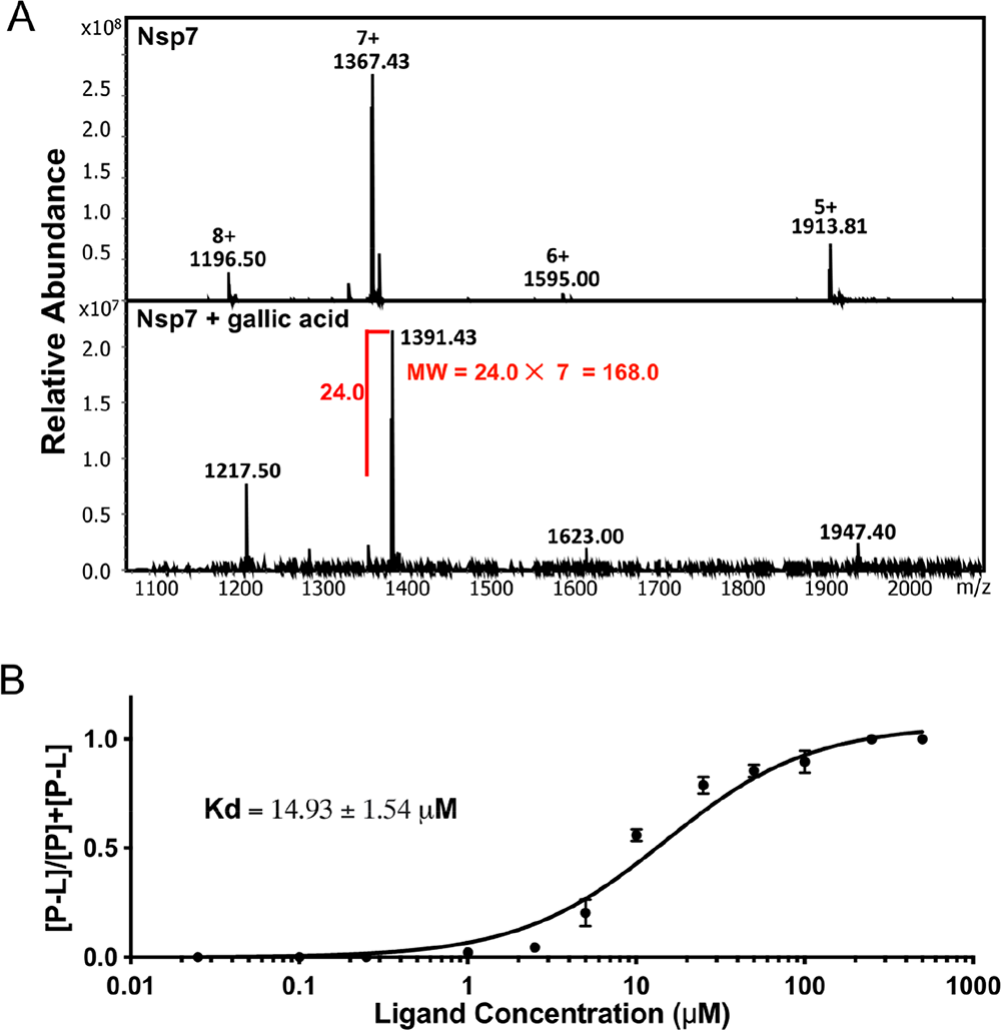
(**A**) Native mass spectra of free nsp7 (top) and nsp7 incubated with gallic acid (bottom) in the dose response study. (**B**) Dose–response of gallic acid binding to nsp7 over a range of ligand concentrations (0.025 μM to 500 μM). Error bars represent the standard deviation of three independent replicates.

The *K*_d_ for gallic acid was measured from the ratio of free and ligand-bound nsp7 in a series of native mass spectra collected over a range of gallic acid concentrations (0.025 μM to 500 μM). The native mass spectra of nsp7 in the dose–response study (Figure 2A) obtained an abundance distribution different from the mass spectra used in the library screening, which may correspond with the two forms of nsp7 structures mentioned in the previous study.^45^ The ratio of the intensity of the protein–ligand peak (P-L) over the sum of the protein (P) and P-L peaks was plotted against the concentrations of gallic acid added to a fixed amount of SARS-CoV-2 nsp7. From this curve, plotted in Figure 2B, a *K*_d_ of 14.93 ± 1.54 μM was determined, indicating that gallic acid bound to nsp7 with micromolar affinity.

### NMR Chemical Shift Perturbation Study of SARS-CoV-2 Nsp7 with Gallic Acid

**Figure 3:**
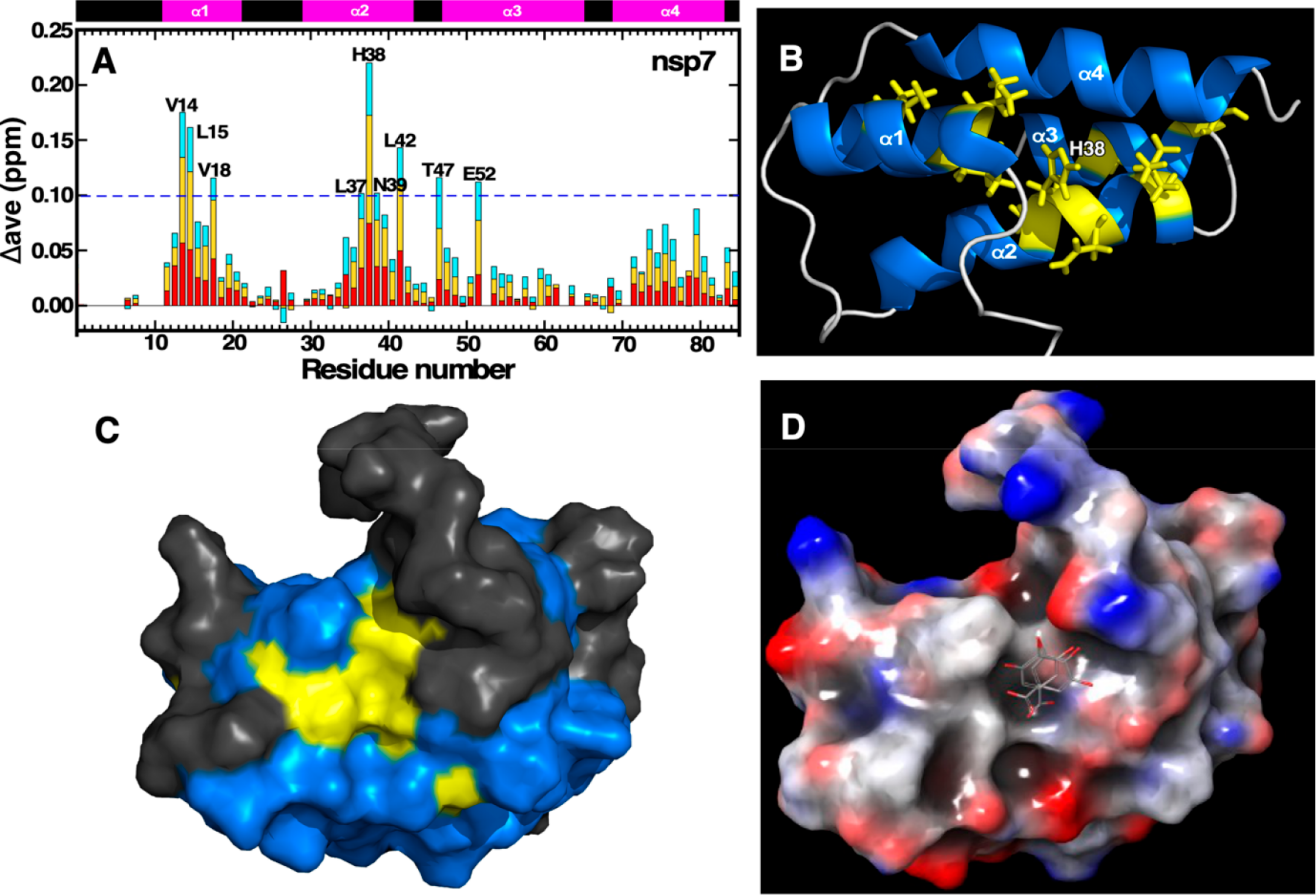
**Mapping of the ligand-binding surface of gallic acid on SARS-CoV-2 nsp7 with an NMR chemical shift perturbation study.** (**A)** Summary of the amide chemical shift perturbations observed in the ^1^H–^15^N HSQC spectrum of ^15^N-labeled SARS-CoV-2 nsp7 at gallic acid:nsp7 molar ratios of 1 (red), 2 (yellow), and 3 (cyan). The average chemical shift change (Δ_ave_ ppm) equals {[(Δ^1^N^H^)^2^ + (Δ^15^N/5)^2^]/2}^1/2^. A schematic representation of the four α-helices (magenta) observed in the solution structure is shown at the top of the plot. (**B**) Cartoon representation of a representative structure from the ensemble of NMR solution structures calculated for SARS-CoV nsp7 (PDB: 2KYS). Residues with a Δ_ave_ greater than 0.1 ppm at the 3:1 gallic acid:nsp7 molar ratio are colored yellow, with the side chains shown. The most perturbed amide resonance, H38, is labeled. (**C**) Solvent-accessible surface rendering of the structure illustrated in panel B, rotated to show the largest perturbed and exposed surface (yellow, L37-N39 and L42) following nsp7 binding to gallic acid. (**D**) Same orientation of the structure illustrated in panel C showing the electrostatic potentials at the solvent-accessible surface (positive regions colored red and negative regions colored blue). A molecule of gallic acid (ball-and-stick rendering) was modeled into the yellow pocket shown in Figure 3C using the Schrödinger docking program, with the location of the gallic acid shown for the top two docking poses.

The primary amino acid sequences of SARS-CoV and SARS-CoV-2 nsp7 are identical aside from a R70K substitution in the sequence encoded by the latter virus.^56^ With MW ≈ 9 kDa, nsp7 is of a size that makes it amenable to study by NMR spectroscopy. Solution NMR structures have been determined for SARS-CoV nsp7 at pH 7.5 and at pH 6.5.^45,57^ More recently, the NMR chemicals shifts for SARS-CoV-2 nsp7 have also been reported.^56^ To corroborate the binding of gallic acid to SARS-CoV-2 nsp7 and to map the ligand-binding surface of gallic acid on nsp7, a chemical shift perturbation study was performed by titrating gallic acid into ^15^N-labeled SARS-CoV-2 nsp7.^58,59^ A summary of this experiment, shown in Figure 3A, indicates that the major chemical shift perturbations (Δ_ave_ > 0.1 ppm) are clustered in α1, the C-terminal of α2, and the N-terminal of α3. These major chemical shift perturbations are mapped onto the solution structure of SARS-CoV nsp7 in Figure 3B (yellow) and show that, except for the two resonances in α3, all the perturbations are along a continuous linear surface spanning α1 and α2 centered roughly at H38, the most perturbed amide resonance overall. A surface rendering of the nsp7 structure with the major perturbations in Figure 3C shows that the largest perturbed solvent-exposed surface is a pocket formed by residues L37-N39 and L42. *In silico* molecular docking using the Schrödinger platform shows that gallic acid comfortably fits into this pocket (Figure 3D).

While the chemical shift perturbations to the ^1^H–^15^N HSQC spectrum of ^15^N-labeled SARS-CoV-2 nsp7 could be followed up to a gallic acid:nsp7 molar ratio of 3:1, at a molar ratio of 3.5:1 amide cross peaks began to lose intensity. Solution structures have been determined for SARS-CoV nsp7 at two pH values and recently for SARS-CoV-2 nsp7 (PDB: 7LHQ).^45,57^ For all three structures, the protein structure was solved as a monomer and there is no crystal structure for nsp7 by itself. There is SAXS evidence that SARS-CoV-2 nsp7 forms multimers in solution.^34^ The native MS experiments show a 1:1 stoichiometry (Figure 2A). Whatever the cause for the loss of signal intensity at the higher gallic acid:nsp7 molar ratios, this loss in NMR signal intensity suggests gallic acid is affecting the protein, and this may be advantageous for inhibiting the function of nsp7.

**Figure 4:**
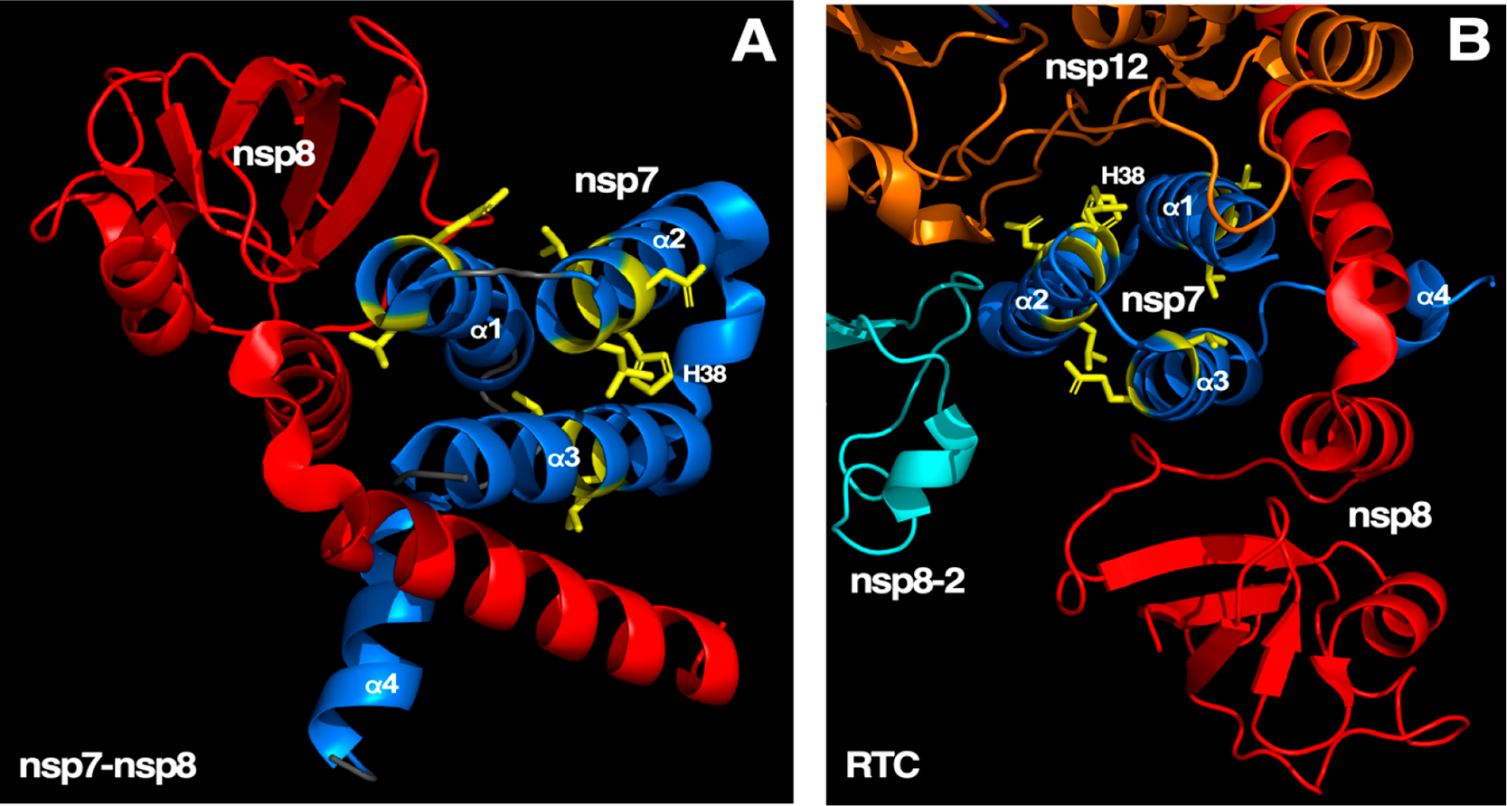
**Position of the nsp7 gallic acid binding pocket in known nsp7 protein–protein complexes.** (**A**) Cartoon representation of the crystal structure (PDB: 6WIQ) of the nsp7-nsp8 heterodimer. Nsp8 is colored red and nsp7 colored blue, with the residues with a Δ_ave_ greater than 0.1 ppm at a 3:1 gallic acid:nsp7 molar ratio colored yellow. The side chains of these perturbed residues are also shown, along with H38 that is in the center of the likely gallic acid binding pocket shown in Figure 3C. The nsp7 gallic acid binding pocket is not at the dimer interface but is solvent exposed. (**B**) Cartoon representation of the cyro-EM structure of the RTC (PDB: 7CYQ). Nsp7 and nsp8 are colored as described in panel A, the second nsp8 structure is colored cyan, and nsp12 is colored orange. The nsp7 gallic acid binding pocket sits in the protein–protein interface between nsp7 and nsp12.

During viral replication there is evidence that nsp7 exists in a dynamic equilibrium with itself, with nsp8, and with the RTC.^34^ To assess if gallic acid binding might disrupt these equilibria, we mapped the chemical shift perturbations observed here for the nsp7 monomer onto the crystal structure of an nsp7-nsp8 heterodimer (PDB: 6WIQ) and the cyro-EM structure of the RTC (PDB: 7CYQ).^33,34^ Further, we assumed that gallic acid physically binds in the pocket centered at H38, as modeled in Figure 3D. For the nsp7-nsp8 complex, illustrated in Figure 4A, this pocket (centered at H38) is solvent exposed and would not directly interfere with the nsp7-nsp8 interface in the nsp7-nsp8 heterodimer. On the other hand, in the RTC this pocket is at the nsp7-nsp12 interface (Figure 4B) and could directly interfere with the stability of the RTC. Hence, disruption of the nsp7-nsp12 interface is another mechanism, in addition to the structural and/or dynamic changes effected in nsp7, by which gallic acid could inactivate the function of nsp7.

**Figure 5:**
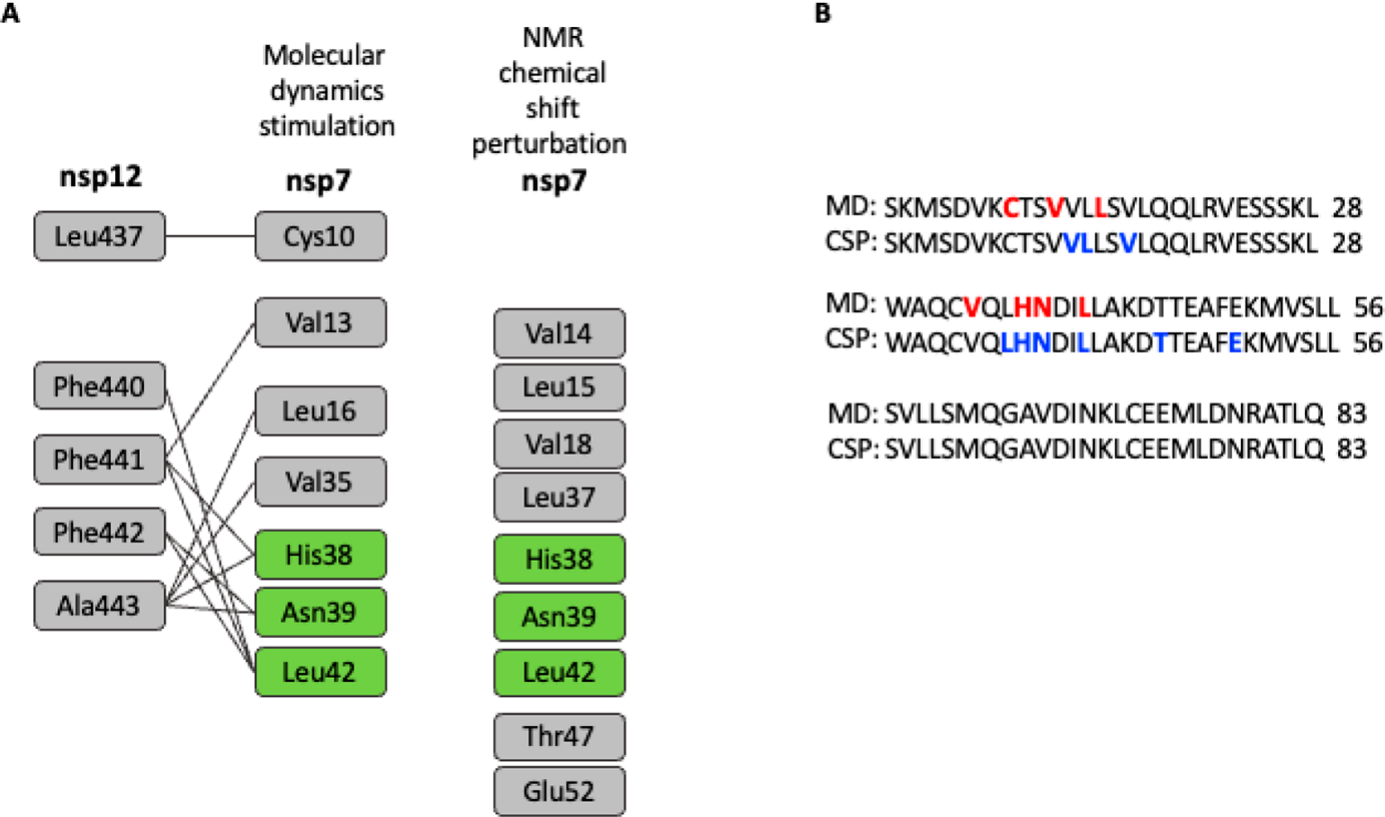
(A) Comparison of interacting interface hotspot residues of nsp7 with nsp12 by molecular dynamics simulation obtained from Sarma et al.^60^ and NMR chemical shift perturbation with gallic acid. Numbering has been adjusted to fit into the chemical shift perturbation construct. (B) Amino acid sequence alignment of nsp7 with hotspots indicated by molecular dynamics simulation from Sarma et al.^60^ (red) and by chemical shift perturbation (blue).

Molecular dynamics (MD) calculations have been run on the interaction between nsp7 and nsp12.^60^ The calculations predicted hotspots for interaction between the two proteins. The NMR chemical shift perturbations for the interaction of gallic acid and nsp7 are similar to the MD-identified hotspots (Figure 5). This indicates that gallic acid could interfere with nsp7-nsp12 complex, thereby inhibiting its function.

## Conclusion

In summary, using native MS, we found that gallic acid—one out of 2400 compounds in an FDA-approved library—was the most potent ligand to bind to SARS-CoV-2 nsp7, a crucial RTC component. Subsequent NMR chemical shift perturbation studies showed gallic acid bound to a surface pocket of nsp7 centered at H38 in α2. Chemical shift perturbations are consistent with hotspots previously identified by molecular dynamics calculations for interaction between nsp7 and nsp12. The identification of the gallic acid binding surface on SARS-CoV-2 nsp7 allows virtual docking and other drug discovery modalities.

## Methods

### Cloning, Expression, and Purification

A codon-optimized expression construct corresponding to the processed protein nsp7 from the *Betacoronavirus* SARS-CoV-2 (2019-nCoV; COVID-19) Wuhan-Hu-1 isolate (Genbank MN908947.3) was synthesized and inserted in the pET28a-TEV vector at the *Nde*I/*Not*I restriction enzymes sites by Genescript (Piscataway, NJ). The recombinant plasmid was then used to transform chemically competent *Escherichia coli* Rosetta BL21(DE3)pLyS cells (Novagen, Millipore Sigma, Burlington, MA) by a heat shock method. The expressed gene product contained a 22 amino acid extension, MGSSHHHHHHSSGENLYFQGHM- at the N-terminus of the native protein to enable protein purification by metal chelation chromatography.^61^ The SSGCID internal ID for the SARS-CoV-2 nsp7 construct is BecoA.18646.a.^62^

Uniformly ^15^N,^13^C-labeled nsp7 was expressed following previously described protocols using minimal medium (Miller) and the antibiotics chloramphenicol and kanamycin.^61^ Nitrogen-15-labeled and unlabeled nsp7’s were expressed using autoinduction protocols with minimal media and LB media, respectively.^63^ Induced cell cultures were harvested by mild centrifugation and then frozen at 193 K. After thawing the frozen pellet, nsp7 was purified with a conventional two-step protocol involving metal chelate affinity chromatography on a 20 mL Ni-Agarose 6 FastFlow column (GE Healthcare, Piscataway, NJ) followed by gel filtration chromatography on a Superdex75 HiLoad 26/60 column (GE Healthcare, Piscataway, NJ). In addition to removing minor impurities, the latter step exchanged nsp7 into the buffer used for the NMR studies: 150 mM NaCl, 50 mM sodium phosphate, 1.0 mM dithiothreitol, pH 6.5. The concentrated protein was diluted 1:1 with a tobacco etch virus (TEV) buffer (50 mM Tris-HCl, 150 mM NaCl, pH 7.8) to a final volume of approximately 1 mL (∼10 mg/mL) and treated overnight at 278 K with 20 μL of TEV protease (0.5 μg/mL, prepared in house) to remove the N-terminal tag. The cleaved protein, which contained three N-terminal scar residues afterward (GHM-), was purified by reapplication on the size exclusion column.

### Nuclear Magnetic Resonance Spectroscopy

The chemical shift perturbation studies were performed under the conditions reported by Johnson et al. because the authors screened a wide range of solution environments to find optimal conditions for the collection of the highest quality NMR data.^45^ Moreover, pH 6.5 is also the pH in the Golgi apparatus inside host cells where viral budding occurs.^64^ While the amide chemical shifts for SARS-CoV were deposited into the Protein DataBank, without a published assigned ^1^H–^15^N HSQC spectrum together with the collection of our chemical shift perturbation data at a lower concentration (0.3 mM versus 2 mM), it was necessary to prepare a ^13^C,^15^N-labeled nsp7 sample and collect two- and three-dimensional NMR backbone assignment data to make unambiguous amide assignments for nsp7.^61^ For the chemical perturbation study, a 0.3 mM solution of ^15^N-labeled nsp7 (300 μL) was prepared along with a 30 mM stock solution of gallic acid in the same NMR buffer. Gallic acid was titrated into the NMR sample in 1.5 μL additions of the stock gallic acid solution with ^1^H–^15^N HSQC spectra collected at 298 K at gallic acid:nsp7 molar ratios of 0, 0.5, 1.0, 1.5, 2.0, 2.5, 3.0, 3.5, and 4.0. All NMR data was collected on an Agilent Inova-600 spectrometer equipped with an HCN-cyroprobe and processed using FELIX (2007). NMR data analyses were performed using NMRFAM-SPARKY (v1.414).

### Compound Library

The FDA-approved drug library used in our screen was obtained from the MicroSource Spectrum Collection at Compound Australia. The library contained 120 pools in DMSO with 20 compounds (250 μmol) in each pool. Each pool was freeze-dried to remove the DMSO, resuspended in 1 μL of methanol, and mixed in with 9 μL of nsp7 protein. The final screening concentration for each compound in the pools was 25 μM.

### Protein Preparation for Native MS

SARS-CoV-2 nsp7 purified in NMR buffer was buffer exchanged into 300 mM ammonium acetate (pH 7) with a NAP-5 column (Cytiva, USA) prior to native MS screening and then diluted to a working concentration of 10 μM. 9 μL of this protein working solution was added to each compound library pool (in 1 μL of methanol) and incubated for 30 min at room temperature prior to native MS screening.

### MS Instrument Control and Acquisition

Experiments were performed on a Bruker SolariX XR 12 T Fourier transform ion cyclotron resonance mass spectrometer (Bruker Daltonics Inc., Billerica, MA) equipped with an automated chip-based nanoelectrospray system (TriVersa NanoMate, Advion Biosciences, Ithaca, NY, USA). Mass spectra were recorded in positive ion and profile modes. Each spectrum was profiled within the mass range of 50 to 6000 *m*/*z*, and a total of 16 scans composed of 1M data points were recorded. Ubiquitin was used as standard at the beginning of each screening. Data were acquired by Solarix control software for Bruker SolariX XR 12T in a Windows operating system. The molecular weight of the binding compounds observed in the spectra was calculated as MW_Ligand_ = Δ*m*/*z* × *z*. The binding of the individual hit compounds was further confirmed in a separate experiment.

### Dose–Response Binding Experiments

Various concentrations of gallic acid were prepared in DMSO by serial dilution (0.25 μM, 1 μM, 2.5 μM, 10 μM, 25 μM, 50 μM, 100 μM, 250 μM, 500 μM, 1000 μM, 2500 μM, and 5000 μM). 1 μL of gallic acid at each concentration was added to each well on a 384-well V-plate microtiter plate (BioCentrix, Carlsbad, CA, USA). The DMSO in each well was removed by freeze-drying (Christ, Osterode am Harz, Germany) ,followed by the addition of 1 μL of MeOH in each well prior to incubation with nsp7 (9 μL, 10 μM in 150 μM, pH 7.2 ammonium actetate). The percentage of protein in a protein–ligand complex observed was calculated using the formula %ligand-bound protein = [P-L]/([P] + [P-L]) × 100%, where [P-L] is the total intensity of the protein–ligand complex and [P] is the total intensity of the apoprotein at a single charge state. GraphPad Prism was used to generate a binding curve by plotting ligand concentration versus the percentage of ligand-bound protein using a non-linear regression (Y = Bmax*X/(*K*_d_ + X)). The percentage of ligand-bound protein in the sample was calculated with three replicates according to the formula described here and averaged between the mass-to-charge ratios of the +5, +6, +7, and +8 charge state peaks.

### Molecular Docking

Molecular docking was conducted using GLIDE as part of the Maestro program (version 12.9.123; Schrödinger, Portland, OR, USA). The ligand-free NMR solution structure of SARS-CoV-2 nsp7 determined at pH 6.5 (PDB: 2KYS) was used as the docking receptor.^45^ The model was prepared according to Schrödinger standard protocols and minimized.^65^ A receptor grid cube (20 Å) was calculated and centered on the H38 side chain. Ellagic and salicylic acids were used as comparators. Standard precision glide docking scores ranged from −5.1 to −1.2 with a mean of −3.25. The top docking score of gallic acid was 1.85σ above the mean score. The top two docking poses show the gallic acid positioned in the H38 pocket near L42. The Research Collaboratory for Structural Bioinformatics Protein DataBank (RSCB PDB) contains two structures of proteins bound to gallic acid: glycogen phosphorylase (4Z5X) and tannin acyl hydrolase (4J0H).^66,67^ In the glycogen phosphorylase complex, gallic acid is bound in a π–π stacking arrangement between a phenylalanine and a tyrosine residue. However, in tannin acyl hydrolase the gallic acid is bound in a solvent-accessible cleft adjacent to residues structurally similar to H38, L42, and E76 of SARS-CoV-2 nsp7.

## Supporting Information Available

Figure S1, carbon isotope distribution of each charge state of nsp7, confirming monomer species (PDF)
