# Supplementary material for: Drug-Repurposing Screening Identifies a Gallic Acid Binding Site on SARS-CoV-2 Non-structural Protein 7
Source: ACS Pharmacol Transl Sci. 2023 Mar 7;6(4):578–86. doi: 10.1021/acsptsci.2c00225 (PMC10111621; doi:10.1021/acsptsci.2c00225)
Supplement: Supplementary file 1 — pt2c00225_si_001.pdf [file pt2c00225_si_001.pdf]

Supplementary information

Drug-Repurposing Screening Identifies a Gallic Acid Binding Site on SARS-CoV-2 non-structural protein (Nsp) 7

Yushu Gu,[†] Miaomiao Liu,[†] Bart L. Staker,[$] Garry W Buchko,[‡+] Ronald J Quinn[†*]

[†] Griffith Institute for Drug Discovery, Griffith University, Brisbane 4111, Australia
[$]Seattle Children's Research Institute, Seattle, Washington 98101, USA
[‡] Earth and Biological Sciences Directorate, Pacific Northwest National Laboratory, Richland, Washington 99354, USA
+ School of Molecular Biosciences, Washington State University, Pullman, WA 99164, USA

*Email: r.quinn@griffith.edu.au

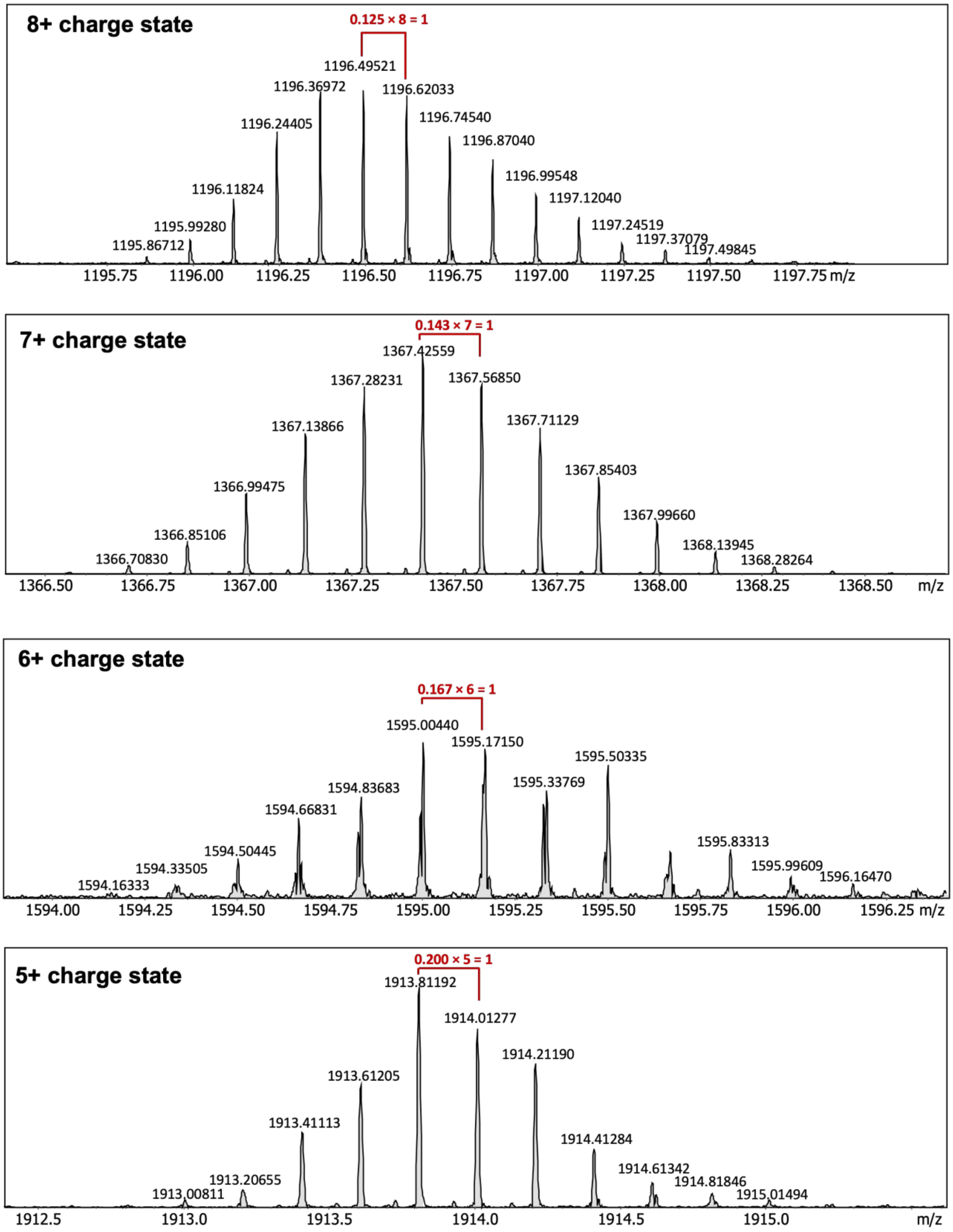

Figure SI 1. Carbon isotope distribution of each charge state of nsp7 confirming monomer species.
